# Land Use Interacts With Climate to Influence Microbial Diversity‐To‐Biomass Ratios Across Europe via Soil Organic Carbon and Nitrogen

**DOI:** 10.1111/mec.17806

**Published:** 2025-05-23

**Authors:** José A. Siles, Alfonso Vera, Maëva Labouyrie, Johan van den Hoogen, Thomas W. Crowther, Ferran Romero, Leho Tedersoo, Carlos García, Arwyn Jones, Panos Panagos, Marcel G. A. van der Heijden, Alberto Orgiazzi, Felipe Bastida

**Affiliations:** ^1^ Department of Soil and Water Conservation and Organic Waste Management Centro de Edafología y Biología Aplicada del Segura‐Consejo Superior de Investigaciones Científicas, CEBAS‐CSIC Murcia Spain; ^2^ Plant‐Soil‐Interactions, Research Division Agroecology and Environment Agroscope Zurich Switzerland; ^3^ Department of Plant and Microbial Biology University of Zurich Zurich Switzerland; ^4^ Department of Environmental Systems Science Institute of Integrative Biology, ETH Zürich Zurich Switzerland; ^5^ Mycology and Microbiology Center University of Tartu Tartu Estonia; ^6^ European Commission, Joint Research Centre (JRC) Ispra Italy; ^7^ European Dynamics, Brussels, Belgium & European Commission, Joint Research Centre (JRC) Ispra Italy

**Keywords:** agroecosystems, continental scale, diversity:biomass, microbial abundance, stress gradient hypothesis

## Abstract

Ecosystem functioning is potentially dependent on the relationships between soil microbial diversity and biomass. Yet, it remains unclear how land use and climate influence these relationships. Here, we (i) analysed relationships and ratios between richness and biomass of bacteria and fungi in ~500 soils across Europe, including three land‐use types (woodlands, grasslands and croplands) and climates (cold, temperate and arid) and (ii) identified the driving factors of changes in richness:biomass (R:B) ratios. Richness and biomass of soil bacteria and fungi followed a unimodal pattern, with a peak in mid‐levels of biomass. This pattern was more evident in bacteria and more clearly exerted by land use than by climate. Bacterial R:B ratios decreased with land use in the following order: croplands > woodlands > grasslands. Fungal R:B ratios decreased as follows: grasslands > croplands > woodlands. Climate was found to interact with land use. In this way, arid climate tended to increase bacterial R:B ratios in the different land uses; however, the agricultural practices associated with croplands seem to buffer this effect. In fungi, the interactive effect of land use and climate was less straightforward than for bacteria. According to our models, soil organic carbon (SOC) and total nitrogen (N) in bacteria and SOC in fungi were identified as the primary predictors of R:B ratios. Therefore, factors related to climate and land‐use change with impact on SOC and N contents are potential disruptors of soil microbial R:B ratios. This study clarifies the diversity:biomass relationships across different land uses and climates.

## 1 Introduction

Soil has been estimated to harbour up to 59% of the total known species on Earth, with bacteria and fungi representing a substantial portion of this diversity (Anthony et al. 2023). Microbes mediate processes essential for ecosystem services, including land productivity, nutrient cycling, degradation of contaminants, pathogen control and climate regulation (Delgado‐Baquerizo et al. 2016; Köninger et al. 2022). The processes mediated by soil bacteria and fungi are dependent on their activity, but also on their diversity and biomass. In this way, soil multifunctionality has proven to be driven by microbial diversity and biomass, among other factors (Delgado‐Baquerizo et al. 2017; Wagg et al. 2014). The interaction between diversity and abundance of soil bacteria and fungi has thus the potential to qualitatively and quantitatively influence ecosystem services and deserves investigation under contrasting land uses and climates. An imbalanced ratio between diversity and biomass may impact soil ecosystem services that rely more heavily on the microbial feature experiencing the greater alteration. For example, Bastida et al. (2021) demonstrated that a diversity‐to‐biomass ratio biased towards diversity negatively impacts a key ecosystem service such as the mineralisation of soil organic matter (SOM).

The unimodal (hump‐shaped) relationship between diversity and biomass is widely regarded as the most common pattern observed in aboveground plant communities (Fraser et al. 2015; Lamont 2024). This pattern suggests that diversity increases with biomass until a peak is reached, after which the relationship becomes negative. Several processes explain this pattern. In ecosystems with low plant biomass, species richness is hypothesized to be limited by abiotic stress. In contrast, in productive ecosystems, competitive exclusion by a small number of highly competitive species is hypothesized to constrain species richness. Other mechanisms explaining the unimodal relationship between plant diversity and biomass are disturbance, evolutionary history and dispersal limitation (Fraser et al. 2015). In recent years, an increasing body of research has expanded its focus to exploring the relationships between diversity and biomass in belowground organisms. For example, Geyer and Barrett (2019) experimentally found a unimodal relationship between microbial biomass carbon and bacterial diversity (Shannon index) in an oligotrophic Antarctic soil amended with increasing amounts of mannitol. More recently, Bastida et al. (2021), using observational data, also reported a unimodal relationship between diversity (richness) and biomass (soil fatty acid contents) of soil bacteria and fungi across globally distributed biomes. Soil carbon (C) content was found to be the main driver of the global variations in bacterial and fungal diversity‐to‐biomass ratios. This explained why the highest richness:biomass (R:B) ratios were found in arid environments, characterised by low C contents, and the lowest ones in C‐rich (and cold) environments.

The recent work of Bastida et al. (2021) advanced our understanding of the diversity‐to‐biomass relationships of soil bacteria and fungi at a global scale and their driving factors. However, it was focused on investigating differences across biomes rather than across land‐use types and contained a very limited number of cropland sites. Consequently, it remains unknown whether the unimodal relationship between diversity and biomass is dependent or not on land‐use type. When comparing woodlands, grasslands and croplands, land use is a key factor influencing soil biological, physical and chemical properties (Foley et al. 2005; Labouyrie et al. 2023). Woodlands are usually regarded as stable ecosystems due to the absence of large‐scale soil disturbance phenomena and typically exhibit greater SOM contents (which also extent into deeper soil layers) than croplands and grasslands due to the low rates of SOM mineralization (Bahram et al. 2018; Romero et al. 2024). This results in woodlands usually harbouring higher levels of soil microbial biomass, particularly in cold climates (Crowther et al. 2019). These increased microbial biomass contents are hypothesized to negatively impact non‐competitive species and reduce bacterial and fungal diversity, leading to stronger unimodal relationships (highly biased towards the right tail) and decreased ratios between diversity and biomass of soil bacteria and fungi. This is explained by the stress gradient hypothesis, which suggests that competition is more intense under non‐stressful conditions, such as those found in woodlands, due to lower nutrient limitations (Hammarlund and Harcombe 2019). Conversely, croplands provide a highly dynamic environment for soil bacteria and fungi due to fertilisation, irrigation, intercropping, vegetation removal and other agricultural practices, with frequent tillage‐mediated habitat homogenization and destruction and rapid nutrient turnover (Szoboszlay et al. 2017). This altogether results in decreased contents of soil organic carbon (SOC) and nitrogen (N) in croplands with respect to woodlands (Siles et al. 2023). In grasslands, agricultural management is usually less intense than in croplands, especially in the extensively managed ones (Labouyrie et al. 2023). However, these ecosystems also create dynamic conditions for soil microorganisms, driven by the dense and short root systems of grasses that grow and die seasonally, resulting in rapid nutrient turnover (Romero et al. 2024). These conditions may represent an environmental stress for soil bacteria and fungi. Under these conditions, according to the stress gradient hypothesis, facilitation and niche partitioning can promote species coexistence and interactions with a decrease in competitive interactions (Bertness and Callaway 1994; Chen et al. 2024; Hammarlund and Harcombe 2019). This may lead to subtler unimodal and increased diversity‐to‐biomass relationships and ratios, respectively, in croplands and grasslands with respect to woodlands. Further, the effects of land use on soil microbial biomass do not stand alone. Climate directly affects environmental conditions such as temperature, precipitation and sunlight hours and also indirectly influences factors like primary production. This, in turn, is reflected in soil physical and chemical properties and can either alleviate or exacerbate the effects of land use on the relationships between diversity and biomass (Redlich et al. 2022; Siles et al. 2023). Arid climate conditions are thus believed to favour relatively higher diversity in comparison to biomass, i.e., higher diversity‐to‐biomass ratios. In agreement, Chen et al. (2024) reported that low SOC and N contents in grasslands and croplands had a positive effect on bacteria alpha diversity at high temperatures.

Using the soil module of the European Commission's Land Use/Cover Area frame statistical Survey (LUCAS Soil, which includes the characterisation of 885 soil samples across Europe), Labouyrie et al. (2023) and Siles et al. (2023) analysed land‐use‐ and climate‐mediated patterns of soil bacterial and fungal diversity (16S rRNA gene and full‐ITS region metabarcoding) and biomass (soil fatty acid content), respectively. Taking advantage of the data generated by these works, we here aimed at (i) confirming the unimodal relationship between diversity and biomass for soil bacteria and fungi across Europe; (ii) elucidating how land use and climate impact the relationships between diversity and biomass and the diversity‐to‐biomass ratios; and (iii) modelling the factors controlling the variations in diversity‐to‐biomass ratios of soil bacteria and fungi under contrasting land uses and climates. We hypothesised that bacterial and fungal diversity‐to‐biomass relationships and ratios would be less pronounced and higher, respectively, in croplands (representing a higher level of land‐use intensification) and grasslands than in woodlands, with arid climate conditions boosting the effect of land use on diversity‐to‐biomass relationship and ratios. Changes in diversity‐to‐biomass ratios were expected to be controlled by soil nutrient contents (mainly SOC), which, in turn, are influenced by land use, climate and plant cover.

## 2 Materials and Methods

### 2.1 Soil Sampling

The soils analysed here form part of the 2018 soil module of the European Commission's Land Use/Cover Area frame statistical Survey, LUCAS Soil (Orgiazzi et al. 2018), the largest cross‐European survey for assessing soil characteristics in relation to land cover and use. Sampling took place in the period from April to December 2018 in 24 European Union member states and the United Kingdom. Soil sampling included three broad land uses: woodlands, grasslands and croplands, and collected soils were classified as belonging to three broad climate regimes: cold, temperate and arid. Climatic classification of each sampled site was conducted according to the Koppen‐Geiger system: arid, MAP < 10 × *P*_threshold_; temperate, *T*_hot_ > 10 and 0 < *T*_cold_ < 18; and cold, *T*_hot_ > 10 and *T*_cold_ ≤ 0. MAP stands for mean annual precipitation. The meaning of *P*_threshold_ varies according to the following rules: if 70% of MAP occurs in winter, then *P*_threshold_ = 2 × MAT; if 70% of MAP occurs in summer, then *P*_threshold_ = 2 × MAT + 28; otherwise, *P*_threshold_ = 2 × MAT + 14. *T*_hot_ stands for temperature of the hottest month, and *T*_cold_ stands for temperature of the coldest month (Peel et al. 2007). Woodlands included broadleaved and coniferous woodlands. Grasslands included sites covered by communities of grasses, grass‐like plants, or forbs. Croplands included both permanent (olive groves, vineyards, etc.) and non‐permanent (wheat, maize, etc.) crops. Each sample was a composite of five subsamples from the top 20 cm of soil: four subsamples orthogonally collected in a 2 m radius from a central subsample. From each sampling site, 500 g of soil were collected, sieved (2 mm) and stored at −20°C at the Joint Research Centre until further processing. The present study selected ~500 LUCAS soils according to the availability of metadata, as well as data on bacterial and fungal diversity and biomass for each soil.

### 2.2 Environmental and Physicochemical Characterisation of Soil Samples

Environmental metadata for soil samples included: mean annual temperature (MAT), mean annual precipitation (MAP), aridity index (i.e., ratio of precipitation to potential evapotranspiration, which increases with more humid conditions and decreases with more arid conditions), and net primary productivity (NPP). These data, as well as those for bulk density, were obtained from available databases as detailed in Siles et al. (2023). Physicochemical characterisation of soil samples included: texture (percentages of sand, silt and clay), pH (in H_2_O), electrical conductivity (EC) and contents of SOC, total N, available phosphorus (P) and extractable potassium (K). These determinations were done following the standard protocols described in Orgiazzi et al. (2018), and data were obtained from the 2018 LUCAS Soil module.

### 2.3 Soil Microbial Diversity

Data on bacterial and fungal diversity (richness) were obtained from the work by Labouyrie et al. (2023). Bacterial communities were characterised by 16S rRNA gene metabarcoding and fungal communities by full‐ITS metabarcoding in 881 LUCAS soils. DNA was extracted using the DNeasy PowerSoil HTP 96 Kit (Qiagen). The primers 515F and 926R (Caporaso et al. 2011; Parada et al. 2016) were used for partial amplification of the 16S rRNA gene, and the primers ITS9mun and ITS4ngsUni (Tedersoo and Anslan 2019) for amplification of the fungal ITS region. More details on DNA extraction and amplification can be found in Labouyrie et al. (2023). Bacterial amplicons were sequenced using an Illumina MiSeq platform with a 2 × 300 paired‐end configuration, and the fungal amplicons were sequenced using a PacBio Sequel II platform. Bioinformatics processing of sequences was done as described in Labouyrie et al. (2023). Briefly, after demultiplexing, quality‐filtering, dereplication and chimera detection, bacterial sequences were grouped into zOTUs (zero‐radius OTUs, also known as amplicon sequencing variants) and fungal sequences into 98%‐identity OTUs. Taxonomic affiliation of the bacterial zOTUs was conducted with the Ribosomal Database Project taxonomic classifier (Wang et al. 2007) against the 16S rRNA training set 16 with a confidence threshold of 0.8. Fungal OTUs were taxonomically identified using BLAST+ ver. 2.11.0 (Camacho et al. 2009) against the UNITE 9.1 database (Abarenkov et al. 2023) with the confidence thresholds specified in Labouyrie et al. (2023). Only those zOTUs classified as belonging to the domain Bacteria and those OTUs classified as Fungi were retained. Sequences were mapped to zOTUs or OTUs at the 97% identity threshold to obtain one zOTU table for the bacterial community and one OTU table for the fungal community. The bacterial zOTU table was normalised to 40,000 sequences, and the fungal OTU table to 500 sequences with the ‐*otutab_rare* command of USEARCH ver. 11 (Edgar 2013). This command normalises a zOTU/OTU table to a fixed number of reads per sample using random subsampling without replacement. Further, bacterial and fungal richness for each sample was calculated using the ‐*alpha_div* command of USEARCH.

### 2.4 Soil Microbial Biomass

Data on biomass of soil bacteria and fungi were obtained from the dataset produced by Siles et al. (2023). Analysis of the soil contents of ester‐linked fatty acid methyl esters (EL‐FAME, hereafter FAME) was used as a proxy for soil microbial biomass. This method has shown a level of reliability comparable to phospholipid fatty acid analysis in measuring soil microbial biomass (Siles et al. 2024). FAME contents were measured following the method developed by Schutter and Dick (2000). Briefly, fatty acids were extracted from microbial cells and released as methyl esters by incubating 3 g of frozen soil with 15 mL 0.2 M methanolic KOH during 1 h at 37°C under periodic shaking. Samples then underwent pH neutralisation with 1 M acetic acid. Subsequently, FAMEs were partitioned into an organic phase by adding 10 mL hexane and vigorous shaking, followed by centrifugation and evaporation of the hexane in a SpeedVac (Labogene). FAMEs were finally resuspended in isooctane and analysed by gas chromatography under the conditions described by Siles et al. (2023). Soil bacterial biomass was quantified by summing the amounts of the fatty acids i15:0, a15:0, i16:0, 16:1ω9c, 10Me16:0, i17:0, cy17:0, 10Me18:0 and cy19:0. For fungal biomass, the amounts of the fatty acids 18:2ω6,9t and 18:2ω6,9c were summed. Data were expressed as nmol FAME g^−1^ dry weight soil.

### 2.5 Richness‐To‐Biomass Ratios of Soil Bacteria and Fungi

For each soil, data on bacterial and fungal richness and bacterial and fungal biomass were matched. After this, 508 (76 woodlands, 114 grasslands and 318 croplands) soils could be characterised for bacterial richness and biomass and 491 soils (82 woodlands, 101 grasslands and 308 croplands) for fungal richness and biomass. Richness and biomass were standardised between 0 and 1 across all the samples for each community to equally weight diversity and biomass (Bastida et al. 2021). Ratios were then calculated by dividing transformed richness by transformed biomass for each soil sample and each community.

### 2.6 Statistical Analyses

Two‐way univariate PERMANOVA (Permutational Analysis of Variance) was used to check whether the bacterial and fungal R:B ratios were significantly (*p* < 0.05) affected by land use and climate and/or their interaction by using the *adonis* function in the R package *vegan* ver. 2.6–6.1 (Oksanen et al. 2013). PERMANOVA results were cross‐validated with the Kruskal–Wallis test using the *kruskal.test* function (data not shown) in base R (ver. 4.4.1., R Core Team). We further performed post hoc analyses to pairwise compare land uses and climates through the application of pairwise permutation tests taking advantage of the *pairwisePermutationMatrix* function in the R *rcompanion* package ver. 2.4.34 (Mangiafico 2017).

Linear and quadratic models were used to evaluate the direction and magnitude of the relationships between diversity and biomass for bacteria and fungi and between the bacterial and fungal R:B ratios and selected predictors. Regressions were calculated with the function *lm* of the R *stats* package ver. 4.3.1 (R Core Team). The best model fit was selected by identifying the regression with the lowest Akaike information criterion (AIC) value. Correlation heatmaps including all the variables in the study were generated using *Hmisc* ver. 5.1–3 (Harrell 2019) and *corrplot* ver. 0.94 (Wei et al. 2017) packages in R.

Random forest analysis was used to identify the significant predictors of the bacterial and fungal richness:biomass ratios across Europe. The list of predictors included land use (a categorical variable representing a gradient of increasing land‐use perturbation in the following order: woodlands < grasslands < croplands (Labouyrie et al. 2023)), environmental conditions (MAP, MAT, aridity index and NPP) and soil physicochemical properties (sand, silt and clay proportions, bulk density, EC, pH and contents of SOC, N, P and K). The importance (i.e., % increase in mean squared error (MSE)) of each predictor and its significance were computed with the R packages *rfPermute* ver. 2.5.2 and *RandomForest* ver. 4.7–1.2 using 500 trees and *nrep* = 1000 (Archer 2022). Each random forest analysis represents the results of five separate runs combined using the tool *combineRP* of *rfPermute*. The performance of each random forest was evaluated using the R package *rfUtilities* ver. 2.1‐5 (Evans and Murphy 2019).

Structural equation modelling (SEM) was used to build a detailed system‐level understanding of the major direct and indirect effects of land use, climate and soil physicochemical properties on the R:B ratios of soil bacteria and fungi. A priori SEMs were constructed where bacterial and fungal R:B ratios were directly influenced by soil physicochemical properties and directly and indirectly by land use, NPP, aridity index and soil sand content. Highly correlated variables (e.g., SOC and N, or sand and clay, Figures S1 and S2) were not simultaneously included in the models to avoid multicollinearity. Among the climate‐related variables, only aridity index was included in the models since it includes information on both MAP and MAT. The R package *piecewiseSEM* ver. 2.3.0.1 (Lefcheck 2016) was used to test how our log‐transformed data fitted our a priori models. Fisher's C statistic and associated *p*‐value and AIC value (Lefcheck 2016) were used to quantify the goodness‐of‐fit of our models.

Data visualisations were conducted using the R package *ggplot2* ver. 3.5.1 (Wickham 2016) and CorelDRAW ver. 2020.

## 3 Results

### 3.1 Richness‐To‐Biomass Relationships and Ratios Across Land Uses and Climates

**FIGURE 1:**
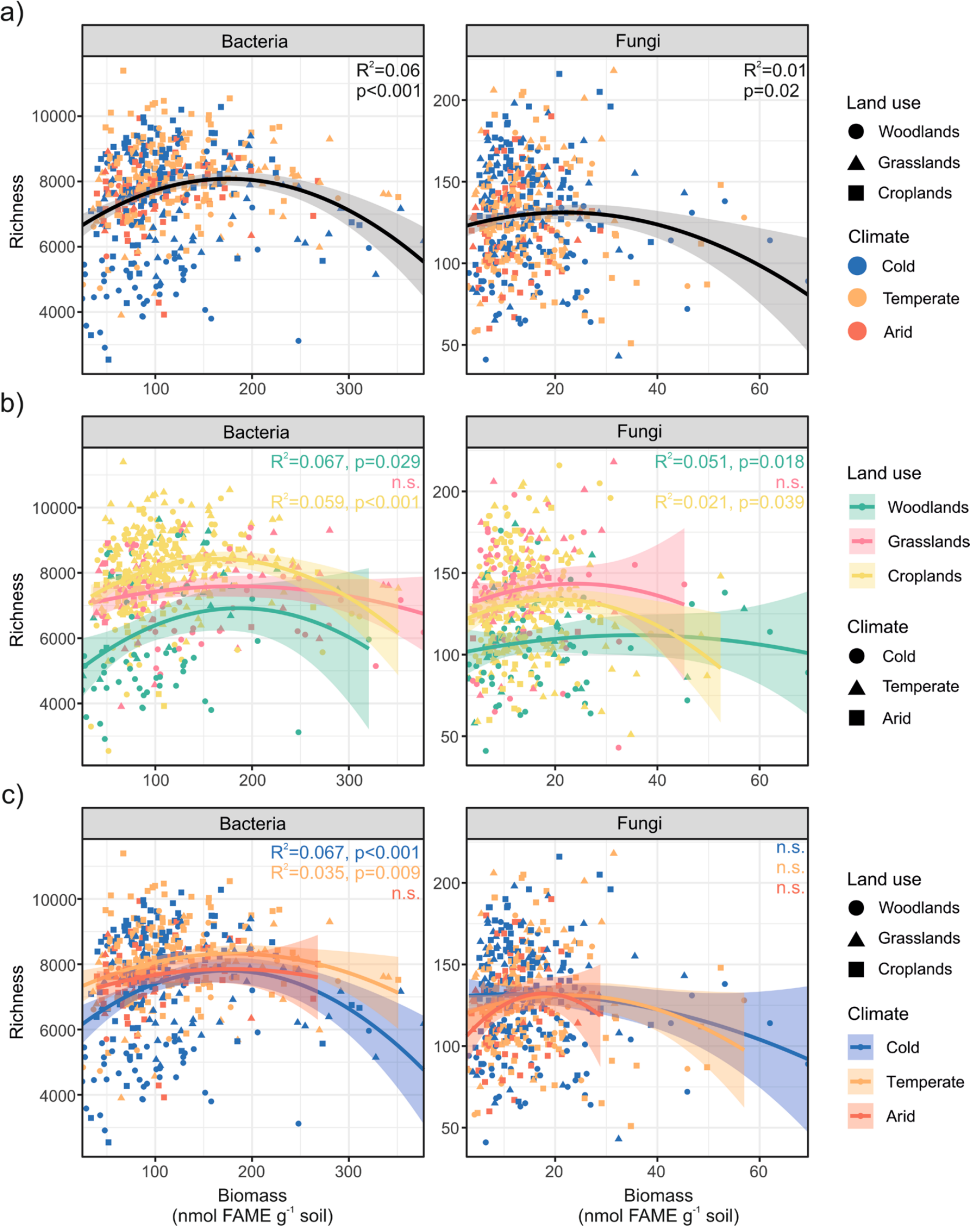
Relationships between richness and biomass for bacteria and fungi as evaluated by regression analyses, considering all soils jointly (a), separately by land use (b), or separately by climate (c). Shaded areas represent 95% confidence intervals for the regression line. Adjusted *R* ^2^ and *p*‐values (*p*) are shown for each regression analysis. n.s., not significant.

Regression analyses showed that diversity (richness) and biomass (soil FAME contents) of both soil bacteria and fungi followed a unimodal pattern across Europe (Figure 1a, Table 1). This trend was also observed within woodland and cropland soils, but not for those under grasslands (Figure 1b, Table 1). Although a significant unimodal relationship was not found between richness and biomass for bacteria and fungi in grassland soils, the quadratic model yielded lower AIC values than the linear one. Significant unimodal associations between bacterial richness and biomass were found in cold and temperate climates, but not under arid conditions (Figure 1c, Table 1). Significant quadratic relationships between fungal richness and biomass were not found in any of the three climates tested, but the AIC values yielded by this model were lower than those returned by the linear model.

**FIGURE 2:**
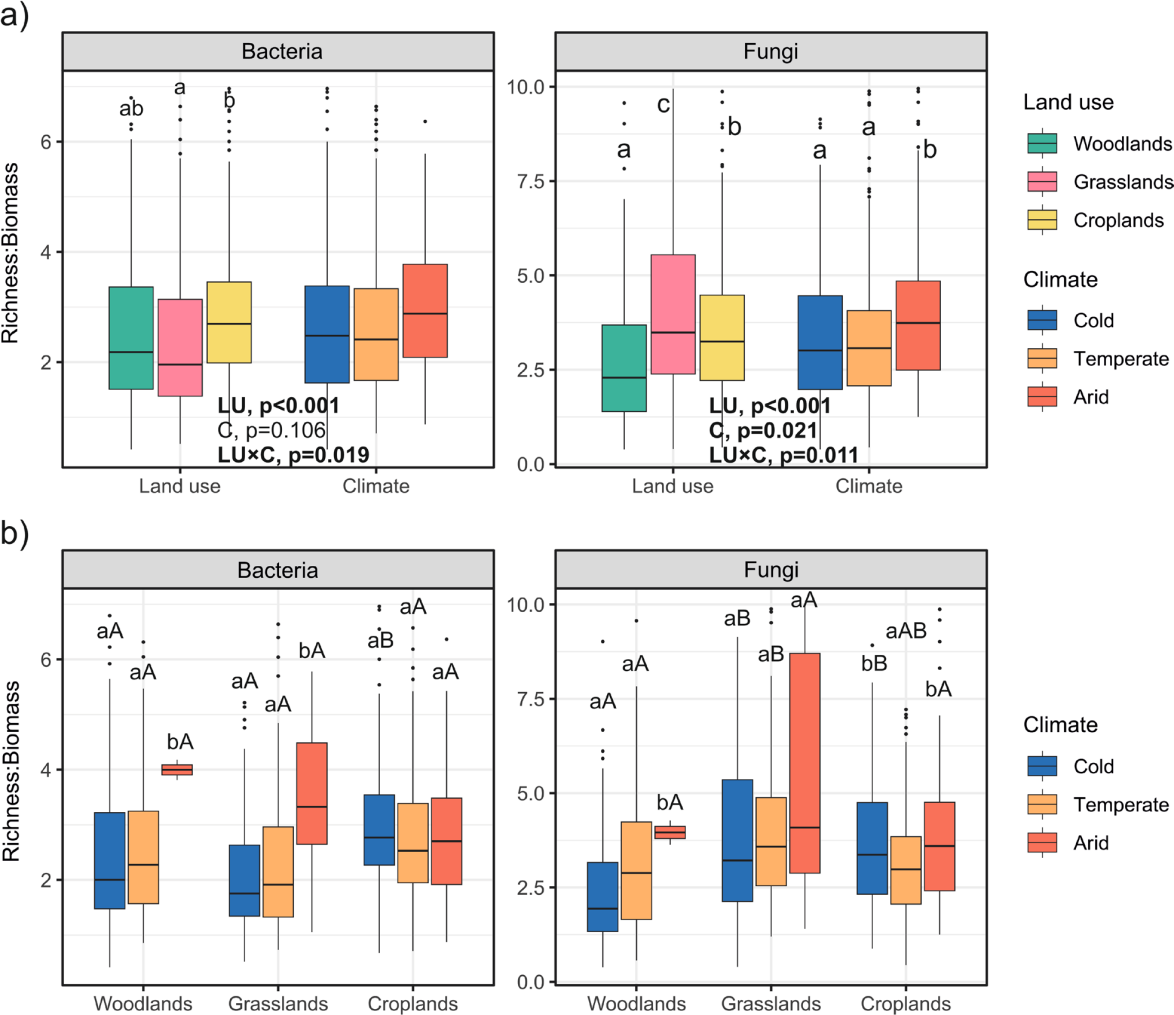
Box plots comparing richness:biomass ratios for bacteria and fungi among land uses and climates (a) and among land uses under arid, temperate and cold climates (b). In (a), *p*‐values (*p*) of two‐way PERMANOVA for the factors land use (LU) and climate (C), and their interaction are shown. Different letters above each box denote significant differences among land uses or climates according to pairwise permutation tests. In (b), different lowercase letters above each box denote significant differences among climates within each land use, and different capital letters denote significant differences among land uses within each climate. The boxes represent the interquartile range (IQR) between the first and third quartiles (25th and 75th percentiles, respectively), and the vertical line inside the box defines the median. Whiskers represent the lowest and highest values within 1.5 times the IQR from the first and third quartiles, respectively. Dots represent outliers.

Bacterial R:B ratios were significantly influenced by land use and the interaction between land use and climate (Figure 2a). Bacterial R:B ratios decreased with land use in the following order: croplands > woodlands > grasslands, with significant differences between croplands and grasslands. Bacterial R:B ratios were, on average, 20% and 27% higher in croplands than in woodlands and grasslands, respectively. Land use, climate and their interaction significantly influenced fungal R:B ratios. They decreased with land use in the following order: grasslands > croplands > woodlands and with climate as follows: arid > temperate = cold. Croplands sustained soil fungal R:B ratios 7% smaller than grasslands and 30% greater than woodlands.

The analysis of the interaction between land use and climate showed that in cropland soils, bacterial R:B ratios did not significantly differ between climates; however, woodland and grassland soils in arid climates sustained higher R:B ratios than those in cold and temperate climates (Figure 2b). In temperate and arid climates, bacterial R:B ratios did not significantly vary with land use, but, in cold climates, cropland soils sustained greater R:B ratios than those under woodlands and grasslands. Soil fungal R:B ratios were higher in arid climates than in cold and temperate ones in woodlands, did not significantly vary with climate in grasslands, and were significantly smaller in temperate climates than in cold and arid conditions in croplands (Figure 2b). In arid climates, fungal R:B ratios did not significantly vary between land uses. In cold and temperate climates, fungal R:B ratios were smaller in woodland soils than in those under grasslands and croplands (Figure 2b).

### 3.2 Factors Driving Richness‐to‐Biomass Ratios Across Europe

**FIGURE 3:**
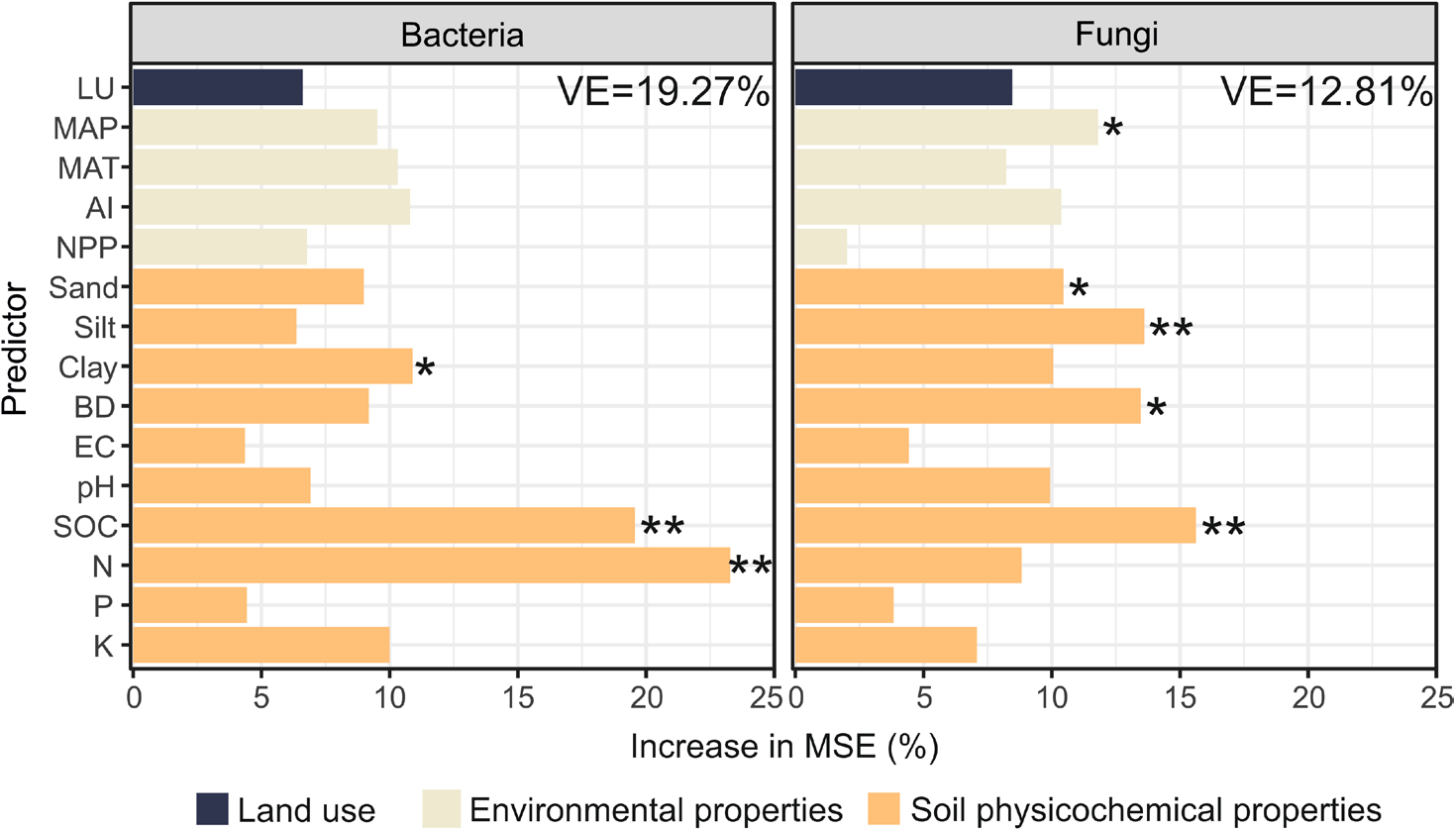
Random forest mean predictor importance (% increase in MSE (mean square error)) of the studied variables as predictors of the variations in bacterial and fungal richness:biomass ratios across land uses and climates. Significance levels are shown at *p < 0.05 and **p < 0.01. Predictors belonging to the same category were represented with the same colour according to the legend. VE, variance explained; AI, aridity index; BD, bulk density; EC, electrical conductivity; K, extractable potassium; LU, land use; MAP, mean annual precipitation; MAT, mean annual temperature; N, soil total nitrogen; NPP, net primary production; P, available phosphorus; Sand, silt and clay, soil sand, silt and clay contents, respectively; SOC, soil organic carbon.

**FIGURE 4:**
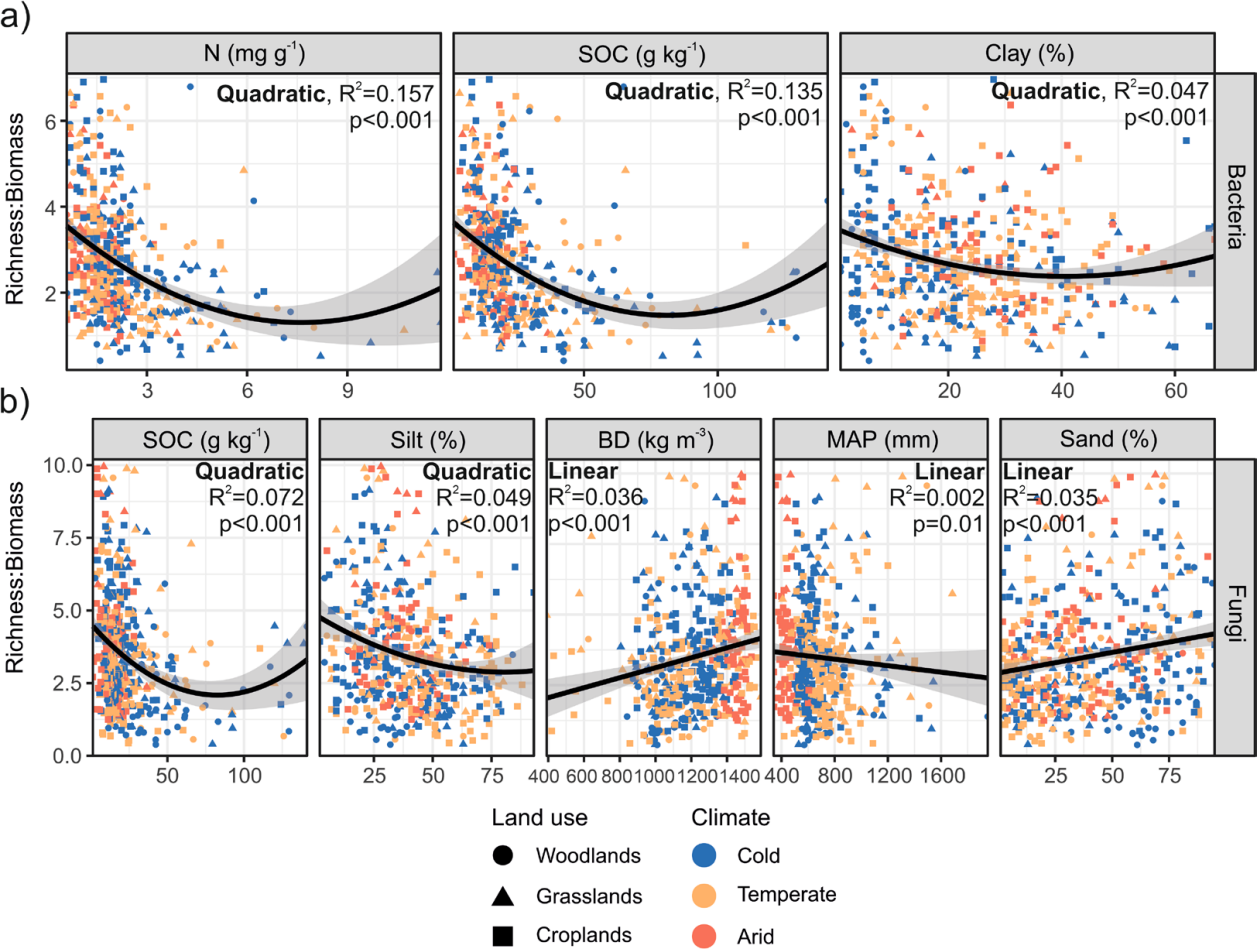
Relationships between bacterial (a) and fungal (b) richness:biomass ratios and selected environmental variables as evaluated by regression analyses. Shaded areas represent 95% confidence intervals for the regression line. The best model (linear or quadratic) fitting each regression is indicated at the top of each figure in the panel. Adjusted *R* ^2^ and *p*‐values (*p*) are shown for each regression analysis. BD, bulk density; MAP, mean annual precipitation; N, soil total nitrogen; Sand, silt and clay, soil sand, silt and clay contents, respectively; SOC, soil organic carbon.

**FIGURE 5:**
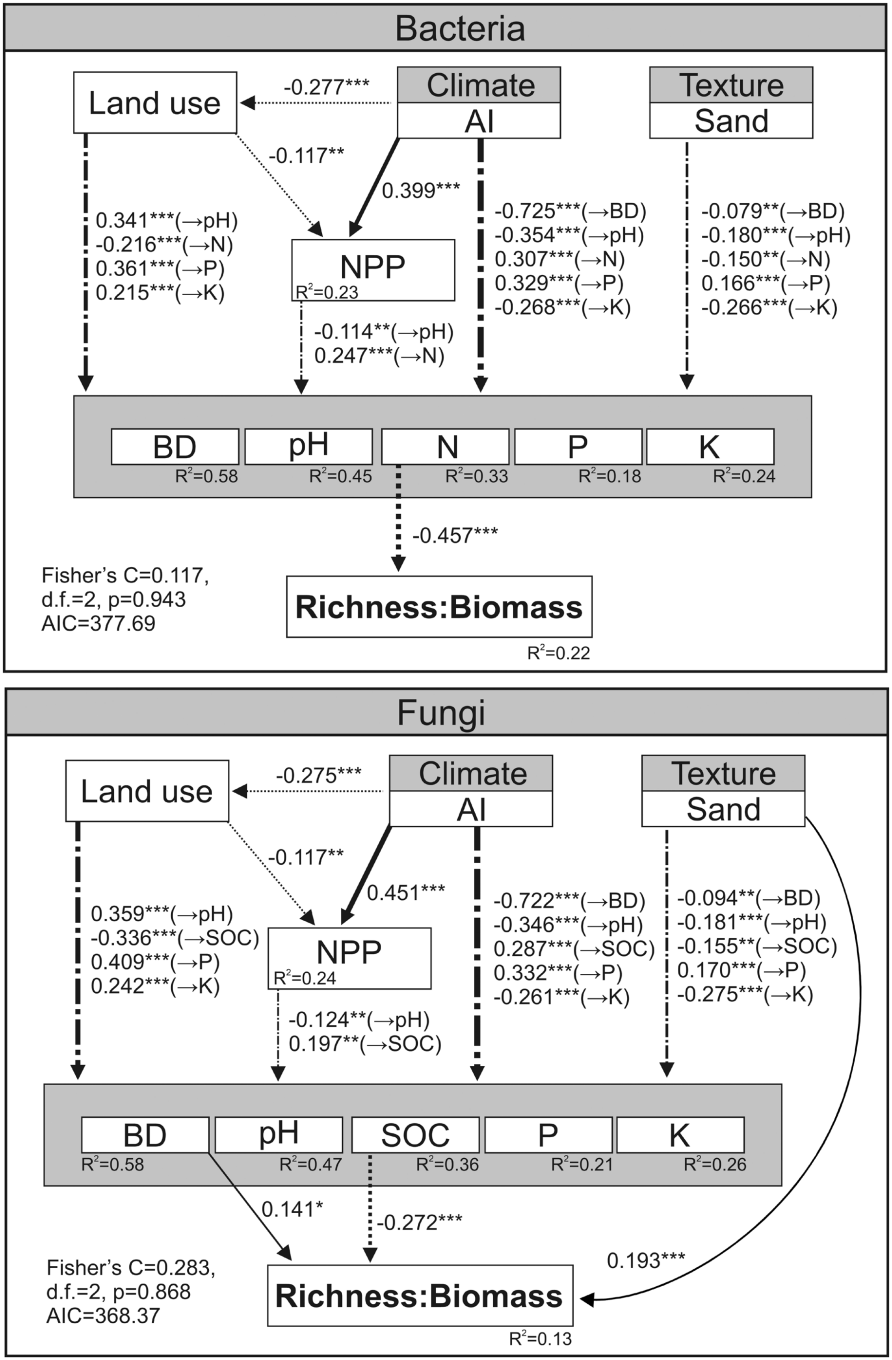
SEM (structural equation modelling) assessing the direct and indirect effects of selected variables on bacterial and fungal richness:biomass ratios. Numbers adjacent to arrows are standardised path coefficients and are indicative of the effect size. Only significant effects (*p* < 0.05) are indicated, and significance levels are shown at *p < 0.05, **p < 0.01 and ***p < 0.001. Continuous, dashed and dash‐dotted arrows indicate positive, negative and mixed relationships, respectively. *R* ^2^ denotes the proportion of variance explained for every response variable by the model. The model's goodness‐of‐fit was evaluated by the Fisher's C statistic and the AIC value. AI, aridity index; BD, bulk density; K, extractable potassium; N, soil total nitrogen; NPP, net primary production; P, available phosphorus; Sand, soil sand content; SOC, soil organic carbon.

Random forest analyses demonstrated that the main (and significant) drivers of land‐use‐ and climate‐driven changes in soil bacterial R:B ratios across Europe are soil contents of N, SOC and clay (Figure 3). These results were confirmed by correlation (Figure S1) and regression analyses (Figure 4a). Bacterial R:B ratios and soil contents of N, SOC and clay were significantly negatively correlated and followed inverse unimodal relationships. The SEM (explaining 22% of the variability in bacterial R:B ratios across Europe) showed that soil N content is the only factor having a direct (and negative) effect on bacterial R:B ratios (Figure 5). In turn, soil N is negatively influenced by land use (an increased land use is represented by croplands) and soil sand content and positively by NPP and aridity. When the SEM was repeated with SOC replaced by N, the same conclusions were reached (Figure S3).

Significant predictors of fungal R:B ratios were SOC, silt, bulk density, MAP and sand content (Figure 3). The relationships between fungal R:B ratios and SOC and silt contents were inverse unimodal, and those between fungal R:B ratios and bulk density, MAP and soil sand content were linear (positive for bulk density and sand, and negative for MAP) (Figures 4 and S2). SEM showed that bulk density, SOC and sand content directly control fungal R:B ratios (Figure 5). In this way, soil sand content (i.e., soil texture) influences fungal R:B ratios directly, but also indirectly by impacting bulk density and SOC content. Bulk density is also controlled by aridity and not by land use or NPP. On the contrary, SOC was directly negatively and positively influenced by land use and NPP, respectively (Figure 5).

## 4 Discussion

Ecosystem services provided by soil are essential for human survival. These services are likely shaped by the relationships between richness and biomass of soil bacteria and fungi, although our understanding of this topic remains limited. Therefore, investigating how richness and biomass of soil bacteria and fungi relate under contrasting land uses and climates across Europe, as well as identifying the factors that drive these changes, is of interest in the current context of global warming and for the European Commission's Common Agricultural Policy (CAP) 2023–2027. The CAP 2023–2027 seeks to promote sustainable agriculture by strengthening food security, supporting farmers' incomes and promoting environmental and climate‐friendly practices, including biodiversity conservation and carbon sequestration (Panagos et al. 2024; Röder et al. 2024). In this scenario, the present cross‐European study surges.

### 4.1 Bacterial Richness‐to‐Biomass Relationships and Ratios Across Europe and Their Driving Factors

Bacterial richness and biomass followed a significant unimodal pattern across the 508 European soils considered in the study. The same pattern was observed after removing 1% of the outliers from our dataset (Figure S4). Our study thus aligns with previous findings, both experimental (Geyer and Barrett 2019) and observational (Bastida et al. 2021), which demonstrate that the relationship between bacterial richness and biomass in soil follows a unimodal pattern, similar to patterns observed in aboveground communities like plants (Fraser et al. 2015). When the three land uses were independently considered, a significant unimodal pattern was found in croplands and woodlands, but not in grasslands. The unimodal relationship was more pronounced (higher quadratic term in the equation model, Table 1) in woodlands than in croplands. Soils under cold and temperate climates further exhibited a significant unimodal relationship between richness and biomass, whereas soils in arid climates did not. When quadratic models of the relationship between richness and biomass in cold and temperate climates were compared, the one for soils under cold climates was found to be more pronounced. All these findings concur with our initial expectations, and they are likely to be explained by the restricting effect that increased levels of bacterial biomass have on bacterial diversity due to the strong competition (with potential exclusion) relationships predicted by the stress gradient hypothesis for nutrient‐rich environments, such as woodlands (in comparison with the other land uses) and cold (in comparison with temperate and arid climate conditions) environments (Bertness and Callaway 1994; Chen et al. 2024).

**TABLE 1:** Quadratic equations derived from the regression analyses in Figure 1 between richness and biomass of soil bacteria and fungi.

	Bacteria	Fungi
All soils	*y* = −0.063*x* ^2^ + 21.97*x* + 6154	*y* = −0.022*x* ^2^ + 0.967*x* + 120
Woodlands	*y* = −0.069*x* ^2^ + 25.893*x* + 4498	*y* = −0.009*x* ^2^ + 0.673*x* + 100
Grasslands	*y* = −0.022*x* ^2^ + 8.388*x* + 6814	*y* = −0.020*x* ^2^ + 1.379*x* + 126
Croplands	*y* = −0.065*x* ^2^ + 21.851*x* + 6612	*y* = −0.040*x* ^2^ + 1.600*x* + 117
Cold	*y* = −0.074*x* ^2^ + 25.627*x* + 5576	*y* = − −0.012*x* ^2^ + 0.295*x* + 130
Temperate	*y* = −0.041*x* ^2^ + 14.852*x* + 6988	*y* = −0.028*x* ^2^ + 1.254*x* + 117
Arid	*y* = −0.031*x* ^2^ + 10.889*x* + 6892	*y* = −0.113*x* ^2^ + 4.034*x* + 96

Despite the observation that the soil bacterial richness‐to‐biomass relationship was sharper curved in woodlands than in croplands, the latter harboured similar R:B ratios to woodlands. Therefore, the lack of significantly increased R:B ratios in croplands with respect to woodlands may be explained by the reduced variability of bacterial richness (standard deviation, 1193) in the former with respect to the latter (1784), as a consequence of the homogenising effect of land‐use intensification associated with agricultural practices on bacterial diversity (Aslani et al. 2024; Rodrigues et al. 2013; Wang et al. 2022). In this way, a growing body of large‐scale research shows that intensified land use boosts microbial diversity but reduces the differences between communities, mainly by negatively affecting specialist taxa adapted to low‐intensity environments (Guerra et al. 2021; Labouyrie et al. 2023). Further, climate was found to be significant for shaping a unimodal pattern between bacterial richness and biomass in cold and temperate conditions; however, it did not directly significantly modify R:B ratios but via its interaction with land use. In this way, our data indicated that arid conditions induced higher bacterial R:B ratios in woodlands and grasslands, but not in croplands. Across both woodlands and grasslands, this phenomenon is explained by the harshening of environmental conditions mediated by aridity (Pawlowska 2024), which potentially makes the cooperation among species more important than competition, with an increase in bacterial specialisation and a relatively higher diversity in comparison with biomass. This finding implies that a drier and warmer climate in Europe may imbalance bacterial R:B ratios towards diversity, impacting ecosystem services driven to a higher extent by bacterial biomass. For example, broad‐spectrum SOM mineralization represents an ecosystem service that is more dependent on bacterial biomass than on diversity, owing to the extensive distribution and functional redundancy of heterotrophic decomposition genes for common substrates among bacterial taxa (Bastida et al. 2021). Conversely, we hypothesise that an imbalance favouring bacterial biomass over diversity may impair ecosystem services like pollutant degradation, as this process relies on a diverse array of enzymes from multiple bacteria taxa (Hernandez‐Raquet et al. 2013). The same principle is hypothesised to apply to other soil processes, including soil disease suppression (Schlatter et al. 2017), bacterial mineralisation of recalcitrant P forms (Siles et al. 2022) and lignin (Wilhelm et al. 2019), and resistance and resiliencies to environmental perturbation (Wagg et al. 2021).

Further, our results showed a lack of increase in R:B ratios with aridity in croplands, which is likely due to the buffering effect of agricultural practices on adverse conditions caused by aridity. Agricultural practices may mitigate nutrient limitations, homogenise soil structure through regular ploughing, optimise soil moisture content via irrigation and foster denser, more consistent plant cover. By alleviating environmental stress, such conditions may reduce the ecological pressure that typically fosters species coexistence and facilitative positive interactions (Chen et al. 2024), mechanisms that are often associated with increased R:B ratios under arid conditions. Unfortunately, the European soil collection used in this study (LUCAS Soil) lacks specific information on the agricultural practices (applied fertilisers, tillage practices, pesticides, irrigation, etc.) applied to each analysed cropland site. This prevented us from digging deeper into the specific factors that limit the aridity‐mediated increase of R:B ratios in croplands.

Our models evidenced that N, SOC and clay (i.e., soil texture) are the dominant drivers of variations in soil bacterial R:B ratios. Further, SEM showed that the only factor having a direct effect on bacterial R:B ratios was N. Our model included N and not SOC because both variables were highly correlated (Spearman *ρ* = 0.87) and because N was a better predictor than SOC according to random forest analysis. When the SEM was repeated substituting SOC for N, the same conclusions were obtained (Figure S3); SOC and N are the sole factors having a direct influence on R:B ratios. Therefore, land use and climate (aridity index) led to changes in R:B ratios by influencing NPP, soil N and SOC contents. The SEM of Bastida et al. (2021) also showed that SOC (also highly correlated with soil N in this study) was the primary factor driving variations in bacterial R:B ratios across global biomes. However, that study also revealed that soil texture and pH directly influence bacterial R:B ratios. These discrepancies may result from differences in the spatial scale and land uses considered. We found a negative correlation between bacterial R:B ratios and SOC and N contents, in concordance with Bastida et al. (2021). However, the inverse unimodal pattern we found between the bacterial R:B ratios and SOC and N would be indicating that in ecosystems with very high contents of N (> 8 mg kg^−1^ soil, Figure 4a) and SOC (> 80 g kg^−1^ soil, Figure 4a), the limiting effect of biomass on diversity is diminished. Further research is thus needed on bacterial richness and biomass links in environments with very high contents of both SOC and N. Our models further evidenced that fine‐textured soils favour decreased R:B ratios, but this effect of soil texture on bacterial R:B ratios is indirectly exerted by modifying soil N contents. This is seen in line with other studies showing that N mineralization rate is lower in fine‐ than coarse‐textured soils because of the physical and chemical protection (Côté et al. 2000; Rong et al. 2021).

### 4.2 Fungal Richness‐to‐Biomass Relationships and Ratios Across Europe and Their Driving Factors

Across all soils in our dataset, fungal richness and biomass followed a unimodal pattern, but this relationship was weaker than that found for bacteria. The same pattern persisted after excluding 1% of the outliers from our dataset (Figure S4). In agreement, Bastida et al. (2021) also reported that the unimodal richness‐to‐biomass relationship was stronger in bacteria than in fungi. Our study also showed that woodland and cropland soils sustained significant unimodal patterns between richness and biomass for fungi. Further, cropland soils presented a more pronounced unimodal relationship and higher ratios between richness and biomass than woodlands (Table 1). We explain this as a consequence of fungi being highly susceptible to agricultural practices. For example, tillage greatly disrupts fungal mycelia, and fungi are more affected than bacteria by dry/wet cycles associated with irrigation since they reside in larger pores (Six et al. 2006). These stress conditions can promote species coexistence and positive interactions with a decrease in competition, leading to increased richness with respect to biomass (Bastida et al. 2021). An imbalanced fungal richness‐to‐biomass ratio towards richness may negatively affect the maintenance of soil physical structure, as this function is largely dependent on the extent and development of fungal mycelial networks (Lehmann and Rillig 2015). Further, grasslands did not sustain a significant unimodal R:B relationship, but they harboured the highest fungal R:B ratios. These findings reveal that stronger unimodal R:B relationships cannot be related with decreased R:B ratios in fungal communities. Additionally, the effect of climate on fungal R:B relationships and ratios was not as clear as that observed for bacteria. This suggests that the mechanisms shaping fungal R:B relationships are complex and that the benign or harsh resource conditions of an environment and the associated proportion of competition or positive interactions between microorganisms do not fully explain how richness and biomass relate.

Random forest identified SOC as the main predictor of land‐use and climate‐mediated changes in fungal R:B ratios across Europe. SEM further showed that SOC was the main factor having a direct effect on fungal R:B ratios. Interestingly, we found that N was not a predictor of fungal R:B ratios, in contrast to bacterial communities. We relate this to soil bacteria being more N‐limited than fungi (Strickland and Rousk 2010; Yu et al. 2022) and soils in our dataset not being probably N‐limiting for fungi (C:N ratios were of 9.2, 10.4 and 15.6 for croplands, grassland and woodlands, respectively (Siles et al. 2023)). The regression analysis showed an inverse unimodal pattern between fungal R:B ratios and SOC contents, meaning that at very high SOC concentrations, fungal R:B ratios increase. We recognise that the number of soils defining this part of the regression is limited, which indicates that further research is necessary to confirm this pattern.

Soil texture (sand and silt contents), bulk density and MAP were identified as predictors of fungal R:B ratios, with the first two variables having a direct effect on the ratios according to our SEM. This evidences that soil physical characteristics control fungal R:B ratios to a higher extent than bacterial ones. Both bulk density and soil sand contents were positively and linearly associated with fungal R:B ratios. Previous studies have reported that both biomass (Chen et al. 2020) and richness (Xia et al. 2020) of fungi are promoted by coarse textured and low compacted soils as a consequence of the preference of these microorganisms to live in soil larger pores due to their filamentous nature (Erktan et al. 2020; Kravchenko et al. 2011). Complementarily, according to our data, fungal richness would be favoured over fungal biomass in soils with high sand contents and bulk density. Large soil pores experience rapid and short fluctuations in resource availability compared to finer pores. For instance, plant debris can easily enter large pores, where it decomposes quickly. Additionally, water drains more rapidly from large pores than from smaller ones due to gravitational pull. These physical characteristics of soil can lead to frequent periods of drought and resource scarcity, creating suboptimal and stressful conditions for fungi (Erktan et al. 2020). These conditions could promote facilitation and niche partitioning through specialisation, leading to the co‐existence of multiple microbial species and further resulting in relatively higher diversity in comparison to biomass (Bastida et al. 2021; Chen et al. 2024). This would explain why fungal R:B ratios increase with soil sand contents and bulk density. In contrast to bacteria, we found that fungal R:B ratios linearly decreased with increasing MAP, which is probably indicating that fungal biomass is favoured over richness under higher soil moisture conditions. We see this in relation with the higher sensitivity of fungi, with respect to bacteria, to drought conditions since they inhabit to a higher extent in soil larger pores (Ullah et al. 2021). Despite this finding, our SEM did not show a direct effect of climate (aridity index) on R:B ratios, but indirectly via bulk density, NPP and SOC. Unfortunately, we cannot compare our results with other studies as, to the best of our knowledge, no previous studies have experimentally investigated how climate directly (i.e., via changes in soil moisture and temperature) influences R:B ratios. In the current context of climate change, this is a key topic that should be addressed.

The close connection between plant communities and mycorrhizal (arbuscular and ectomycorrhizal) fungi is well known, as well as its sensitivity to land‐use change and climate conditions (Labouyrie et al. 2023; Zhou et al. 2024). Further, Romero et al. (2024) found that soil mycorrhiza positively relates with NPP across Europe. We thus expected to find a significant and direct effect of NPP on fungal R:B ratios. However, contrary to our initial expectations, our SEM did not report such direct association; NPP indirectly influenced fungal R:B ratios via changing SOC contents. This aligns with the assumption that soil physical and chemical parameters act as a bridge connecting aboveground and belowground communities (Wardle et al. 2004).

### 4.3 Study Limitations and Future Perspectives

On one hand, our models greatly advanced our understanding of the factors driving changes in bacterial and fungal R:B ratios mediated by land use and climate; however, they showed a rather low explanatory power (i.e., low *R* ^2^ values), especially for fungi. The models were likely found to be statistically significant due to the large sample size; however, their limited explanatory power reflects the high variability of data within each land use. Therefore, future studies should include multiple sampling points within the same location to account for micro‐scale variability in soil microbial communities (Labouyrie et al. 2023). We also attribute the considerable unexplained variance in our models to the absence of key predictors that likely influence R:B ratios. For instance, there might be uncaptured variations in soil moisture (Wan et al. 2021). Furthermore, we were unable to account for factors such as land management practices and disturbance history (Allan et al. 2014; de Vries et al. 2012), nor the potential influences of the surrounding landscape (Le Provost et al. 2021). Future studies should investigate the influence of these factors on microbial R:B ratios and examine how imbalances in these ratios may affect soil functioning. On the other hand, soil microbial diversity can be assessed using a variety of metrics (Roswell et al. 2021). We here used richness rather than other diversity metrics, such as the Shannon index, because (a) this study aimed at complementing the work of Bastida et al. (2021), which also employed richness, and (b) richness is usually more sensitive than the Shannon index in accounting for the rare community (Roswell et al. 2021). Nonetheless, when we repeated the analyses using the Shannon index instead of richness to measure diversity, the results were largely equivalent (Figure S5). We here determined soil contents of EL‐FAME as a proxy for soil microbial biomass. According to Siles et al. (2024), similar results are expected when using other common techniques for quantifying soil microbial biomass, such as quantitative PCR or phospholipid fatty acid analysis. We are aware that these techniques serve only as a proxy for actual soil microbial biomass; however, they offer faster and more precise quantification of bacterial and fungal abundances compared to methods that measure actual microbial biomass (e.g., soil fumigation with chloroform and subsequent C extraction and quantification).

## 5 Conclusions

Richness and biomass of bacteria and fungi followed a unimodal pattern in soils across Europe, with this relationship being stronger in bacteria. Land use was a stronger factor than climate in inducing unimodal relationships between diversity and biomass for bacteria and fungi. Bacterial and fungal richness‐to‐biomass ratios significantly changed with land use and these shifts were, in turn, influenced by climate. For bacteria, arid climate tends to increase R:B ratios across land uses; however, the agricultural practices associated with croplands seem to buffer this effect. The observed patterns in fungal R:B ratios across land uses and climates were not as straightforward as those observed for bacteria, probably because the first ones are regulated by even more complex relationships between below‐ and above‐ground communities than the second ones. Overall, our study demonstrates that land use interacts with climate to drive microbial R:B ratios across Europe. N and SOC were identified as the primary predictors with a direct influence on bacterial R:B ratios, while fungal R:B ratios were directly governed by SOC. Therefore, factors related to climate change (rising in temperatures, drought, etc.) or land‐use change with impact on SOC and N contents are potential disruptors of microbial R:B ratios, leading to implications for ecosystem functioning. In this context, climatic and land‐use change projections may help identify European areas more susceptible to this phenomenon and design monitoring schemes. Further research on this topic should address how specific agricultural practices (tillage, fertilisation, monoculture, etc.) impact microbial diversity‐to‐biomass relationships and ratios.

## Author Contributions

**José A. Siles:** conceptualisation, formal analysis, visualisation, writing – original draft preparation. **Alfonso Vera:** investigation, writing – review and editing. **Maëva Labouyrie:** investigation, writing – review and editing. **Johan van den Hoogen:** investigation, writing – review and editing. **Thomas W. Crowther:** investigation, writing – review and editing. **Ferran Romero:** investigation, writing – review and editing. **Leho Tedersoo:** funding acquisition, investigation, writing – review and editing. **Carlos García:** funding acquisition, writing – review and editing. **Arwyn Jones:** project administration, writing – review and editing. **Panos Panagos:** project administration, writing – review and editing. **Marcel G. A. van der Heijden:** funding acquisition, writing – review and editing. **Alberto Orgiazzi:** project administration, writing – review and editing. **Felipe Bastida:** funding acquisition, writing – review and editing.

## Conflicts of Interest

The authors declare no conflicts of interest.

## Benefit‐Sharing Statement

Benefits from this research accrue from providing scientific information relevant to microbial ecology and environmental sciences, as well as by sharing our data and results on public databases as described above.

## Supporting information


Data S1.

## Data Availability

DNA metabarcoding sequences used in this study to calculate richness have been deposited in the Sequence Read Archive (SRA) database under Bio‐Project IDPRJNA952168. Raw data and metadata used for bacterial community analyses are available at https://doi.org/10.6084/m9.figshare.28920701, while those used for fungal community analyses are available at https://doi.org/10.6084/m9.figshare.28920773. R scripts required to reproduce the figures and statistical analyses in this article are available at https://github.com/J‐Siles/Microbial‐Richness‐Biomass‐ratios‐across‐Europe.
