# Supplementary material for: Land Use Interacts With Climate to Influence Microbial Diversity‐To‐Biomass Ratios Across Europe via Soil Organic Carbon and Nitrogen
Source: Mol Ecol. 2025 May 23;34(20):e17806. doi: 10.1111/mec.17806 (PMC12530285; doi:10.1111/mec.17806)

MOLECULAR ECOLOGY

Supplemental Information for:

Land use interacts with climate to influence microbial diversity-to-biomass ratios across Europe via soil organic carbon and nitrogen

José A. Siles[1,*], Alfonso Vera[1], Maëva Labouyrie[2,3], Johan van den Hoogen[4], Thomas W. Crowther[4], Ferran Romero[2], Leho Tedersoo[5], Carlos García[1], Arwyn Jones[6], Panos Panagos[6], Marcel G. A. van der Heijden[2,3], Alberto Orgiazzi[7], Felipe Bastida[1]

[1]Department of Soil and Water Conservation and Organic Waste Management, Centro de Edafología y Biología Aplicada del Segura-Consejo Superior de Investigaciones Científicas, CEBAS-CSIC, Murcia, Spain. [2]Plant-Soil-Interactions, Research Division Agroecology and Environment, Agroscope, Zurich, Switzerland. [3]Department of Plant and Microbial Biology, University of Zurich, Zurich, Switzerland. [4]Department of Environmental Systems Science, Institute of Integrative Biology, ETH Zürich, Zurich, Switzerland. [5]Mycology and Microbiology Center, University of Tartu, Tartu, Estonia. [6]European Commission, Joint Research Centre (JRC), Ispra, VA, Italy. [7]European Dynamics, Brussels, Belgium & European Commission, Joint Research Centre (JRC), Ispra, VA, Italy

(*) Corresponding author: josesimartos@gmail.com

Table of Contents:

**Fig. S1**	Page 2
**Fig. S2**	Page 3
**Fig. S3**	Page 4
**Fig. S4**	Page 5
**Fig. S5**	Page 6

Fig. S1. Heatmap showing significant ($p < 0.05$) Spearman correlations between all the variables included in the bacterial community dataset (n=508). MAP = mean annual precipitation. MAT = mean annual temperature. AI = aridity index. NPP = net primary production. Sand, silt, and clay = soil sand, silt, and clay contents, respectively. BD = bulk density. EC = electrical conductivity. SOC = soil organic carbon. N = soil total nitrogen. P = available phosphorus. K = extractable potassium.

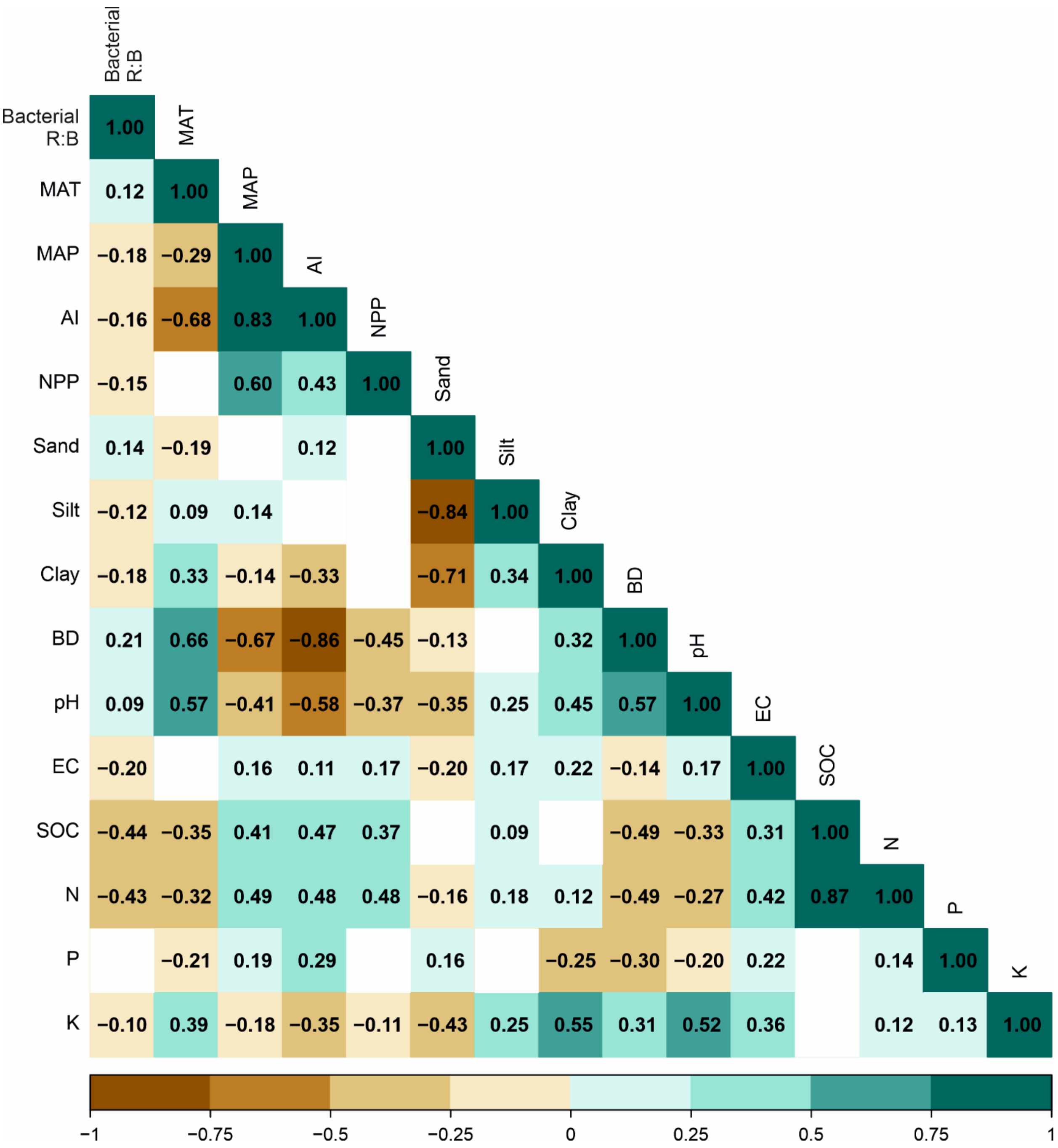

Fig. S2. Heatmap showing significant ($p < 0.05$) Spearman correlations between all the variables included in the fungal community dataset (n=491). MAP = mean annual precipitation. MAT = mean annual temperature. AI = aridity index. NPP = net primary production. Sand, silt, and clay = soil sand, silt, and clay contents, respectively. BD = bulk density. EC = electrical conductivity. SOC = soil organic carbon. N = soil total nitrogen. P = available phosphorus. K = extractable potassium.

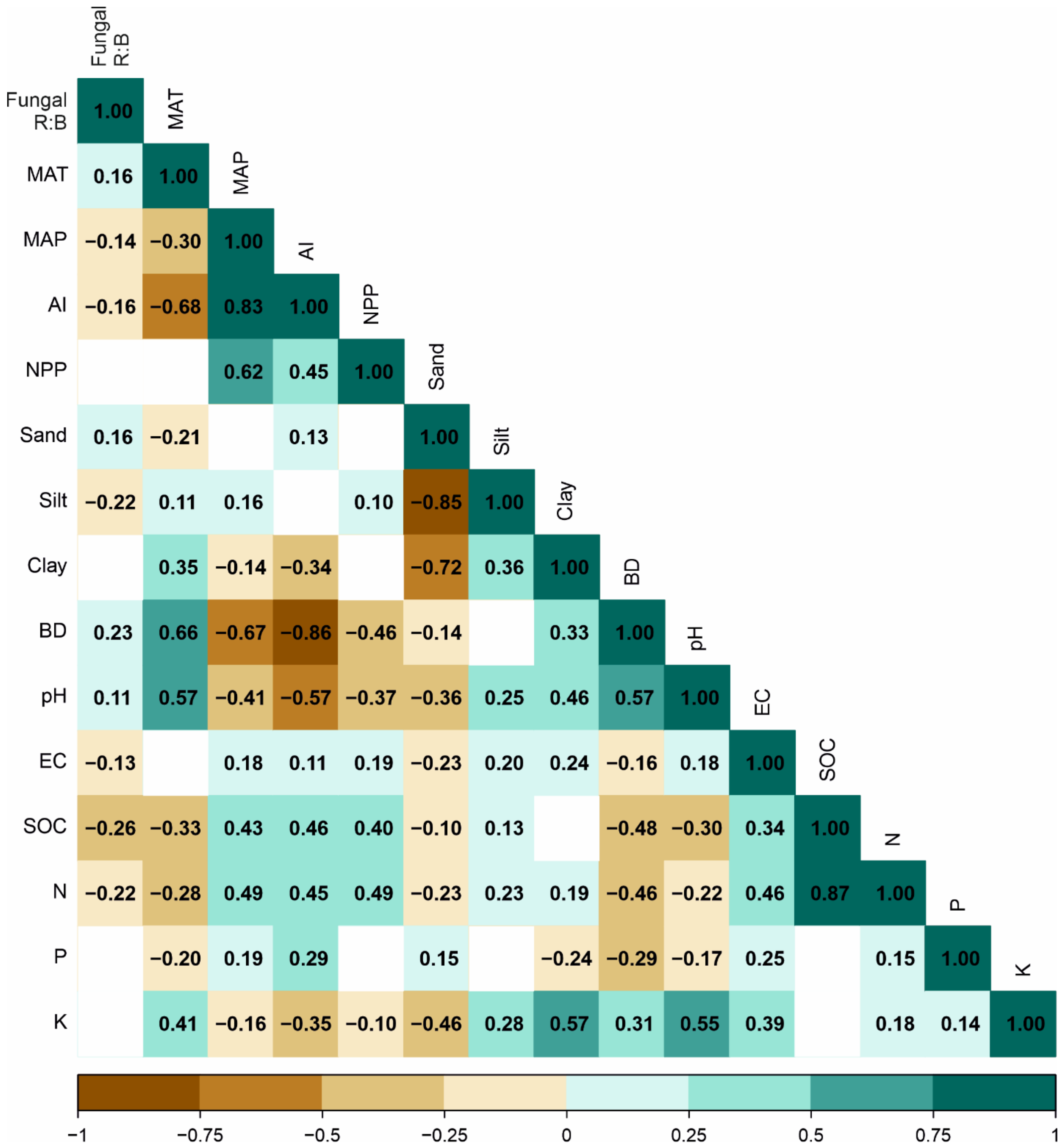

Fig. S3. SEM (structural equation modeling) assessing the direct and indirect effects of selected variables on bacterial richness:biomass ratios. Numbers adjacent to arrows are standardized path coefficients and are indicative of the effect size. Only significant effects ($p < 0.05$) are displayed, and significance levels are shown at *p < 0.05, **p < 0.01, and ***p < 0.001. Continuous, dashed, and dash-dotted arrows indicate positive, negative, and mixed relationships, respectively. R^2 denotes the proportion of variance explained for every response variable by the model. Model's goodness-of-fit was evaluated by the Fisher's C statistic and the AIC value. AI = aridity index. Sand = soil sand content. NPP = net primary production. BD = bulk density. SOC = soil organic carbon. P = available phosphorus. K = extractable potassium.

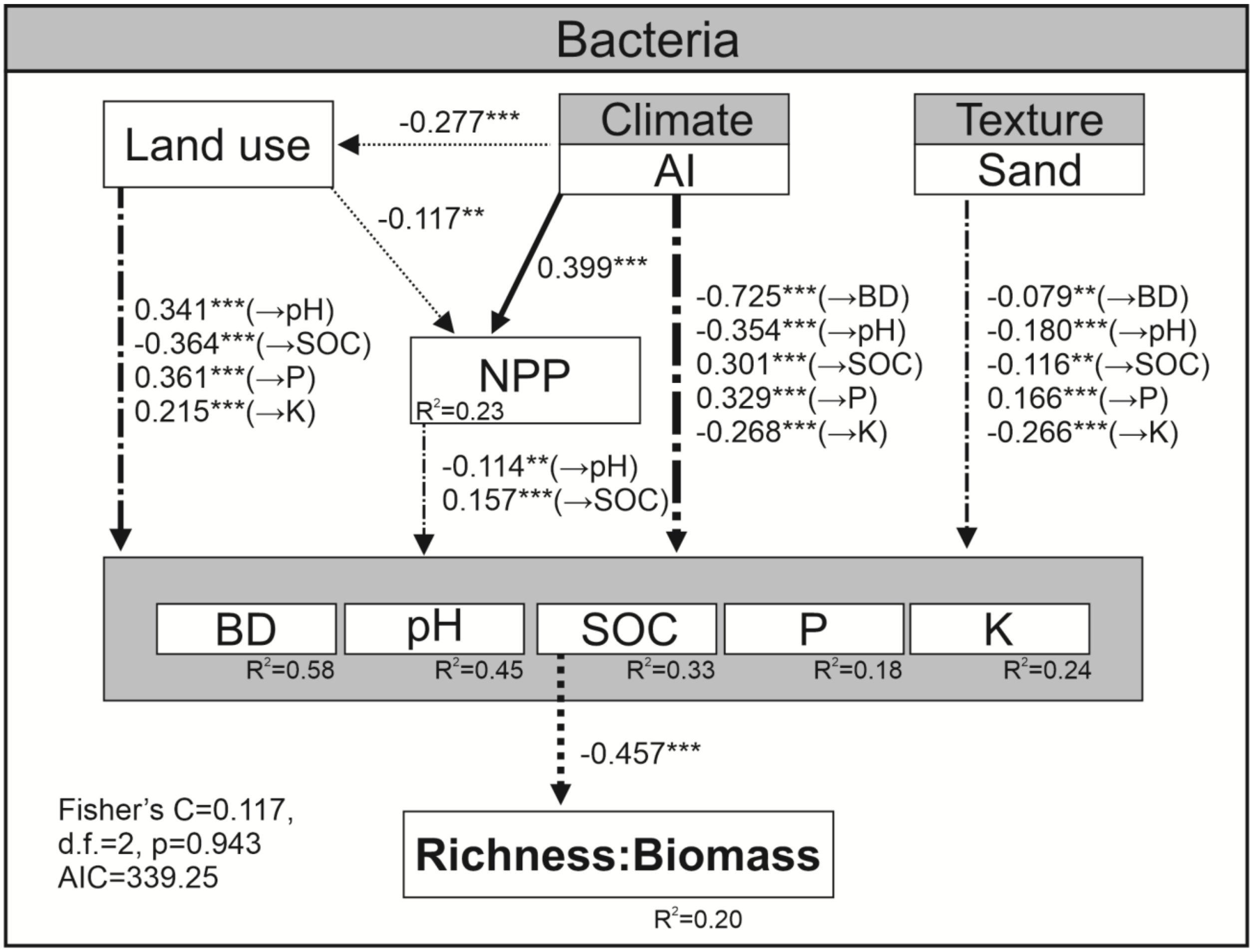

Fig. S4. Relationships between richness and biomass for bacteria and fungi as evaluated by regression analyses after removing 1 % outliers in Fig. 1. Shaded areas represent 95 % confidence intervals for the regression line. Adjusted R^2 and p-values (p) are shown for each regression analysis.

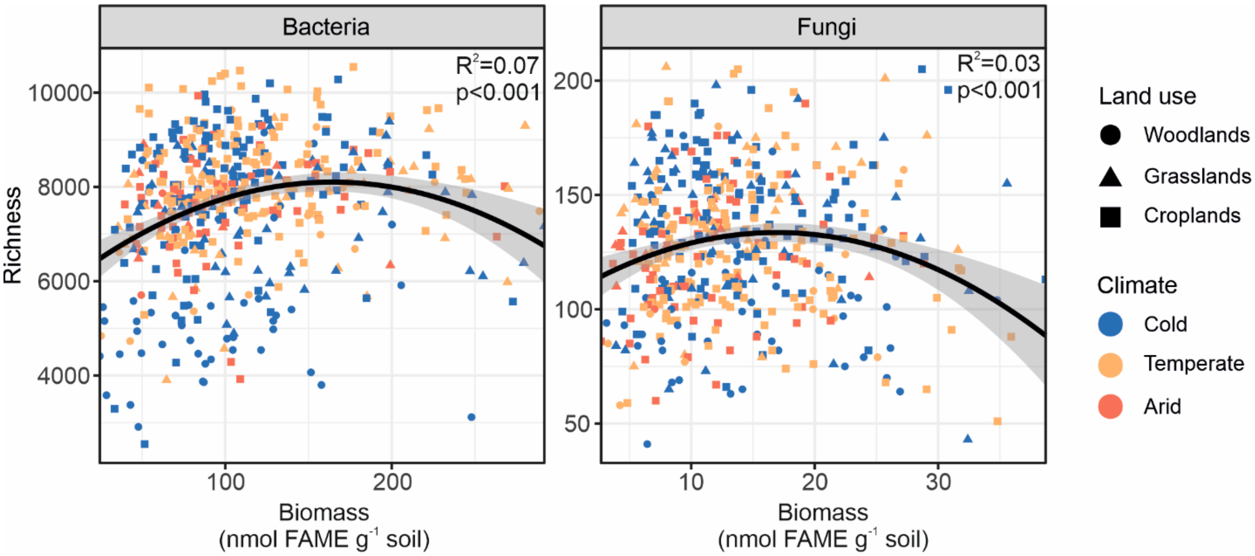

Fig. S5. (a) Relationships between Shannon index and biomass for bacteria and fungi as evaluated by regression analyses. Shaded areas represent 95 % confidence intervals for the regression line. Adjusted R^2 and p-values (p) are shown for each regression analysis. (b) Box plots comparing Shannon index:biomass ratios for bacteria and fungi among land uses and climates. P-values (p) of two-way PERMANOVA for the factors land use (LU) and climate (C), and their interaction are shown. Different letters above each box denote significant differences among land uses or climates according to pairwise permutation tests. The boxes represent the interquartile range (IQR) between the first and third quartiles (25th and 75th percentiles, respectively), and the vertical line inside the box defines the median. Whiskers represent the lowest and highest values within 1.5 times the IQR from the first and third quartiles, respectively. Dots represent outliers. Shannon index was calculated with the command *-alpha_div* of USEARCH. Shannon index and biomass were standardized between 0 and 1 across all the samples for each community to equally weight diversity and biomass.

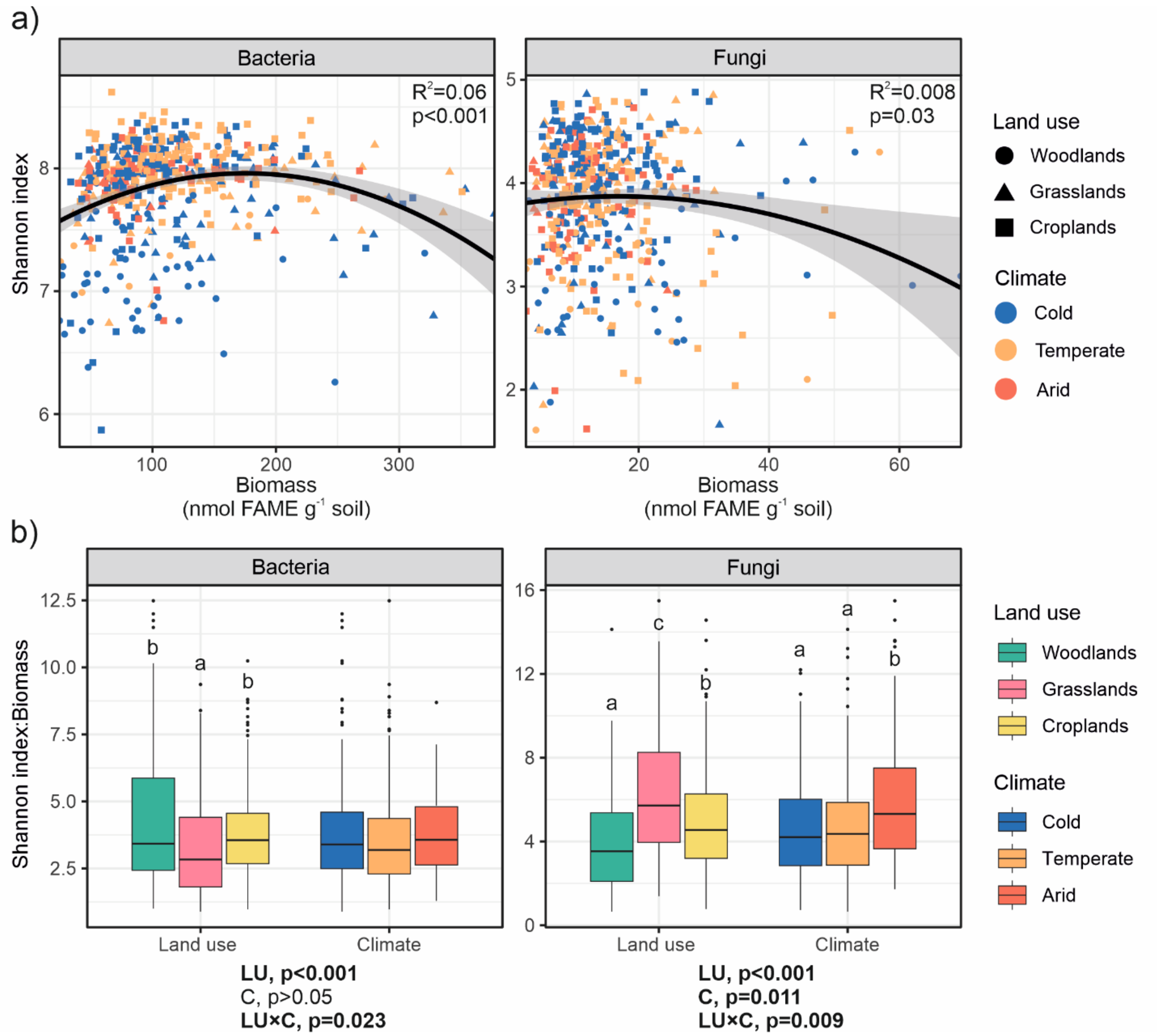

Supplement: Supplementary file 1 — Data S1. [file MEC-34-e17806-s001.pdf]
